# Microvesicle Shedding and Lysosomal Repair Fulfill Divergent Cellular Needs during the Repair of Streptolysin O-Induced Plasmalemmal Damage

**DOI:** 10.1371/journal.pone.0089743

**Published:** 2014-02-21

**Authors:** Alexander P. Atanassoff, Heidi Wolfmeier, Roman Schoenauer, Andrea Hostettler, Avi Ring, Annette Draeger, Eduard B. Babiychuk

**Affiliations:** 1 Department of Cell Biology, Institute of Anatomy, University of Bern, Bern, Switzerland; 2 Department of Protection, Norwegian Defence Research Establishment, Kjeller, Norway; University of Toledo, United States of America

## Abstract

Pathogenic bacteria secrete pore-forming toxins that permeabilize the plasma membrane of host cells. Nucleated cells possess protective mechanisms that repair toxin-damaged plasmalemma. Currently two putative repair scenarios are debated: either the isolation of the damaged membrane regions and their subsequent expulsion as microvesicles (shedding) or lysosome-dependent repair might allow the cell to rid itself of its toxic cargo and prevent lysis. Here we provide evidence that both mechanisms operate in tandem but fulfill diverse cellular needs. The prevalence of the repair strategy varies between cell types and is guided by the severity and the localization of the initial toxin-induced damage, by the morphology of a cell and, most important, by the incidence of the secondary mechanical damage. The surgically precise action of microvesicle shedding is best suited for the instant elimination of individual toxin pores, whereas lysosomal repair is indispensable for mending of self-inflicted mechanical injuries following initial plasmalemmal permeabilization by bacterial toxins. Our study provides new insights into the functioning of non-immune cellular defenses against bacterial pathogens.

## Introduction

Bacteria secrete toxins which form trans-membrane pores in the plasmalemma of host cells [1], [2]. The formation of the pores results in plasmalemmal permeabilization followed by an influx of extracellular and an efflux of intracellular components eventually leading to cell lysis. Since the efflux of intracellular components, which include lytic enzymes, can be detrimental to the surrounding non-injured cells and can also lead to the uncontrolled activation of immune responses, cell lysis must be prevented by any means. In nucleated mammalian cells this is achieved by the process of plasmalemmal repair [3], [4], [5], [6].

It is believed that the isolation of the damaged membrane regions and their subsequent extracellular release as microvesicles or intracellular internalization by lysosome-plasmalemmal fusion and endocytosis allows the cell to rid itself of toxic cargo and re-establish its homeostasis [7], [8], [9], [10], [11].

Lysosomal repair is instrumental in the resealing of mechanically-induced plasmalemmal lesions where lysosomes provide membrane material, which is required for the resealing of mechanically-damaged plasmalemma [6], [8]. This mode of repair might also be involved in the repair of trans-membrane pores formed by the bacterial toxin, streptolysin O (SLO). A currently discussed scenario implies that Ca^2+^-dependent fusion between lysosomes and the SLO-damaged plasmalemma leads to the exposure of the sphingomyelin-rich outer leaflet of the plasmalemmal lipid bilayer to the lysosomal acid sphingomyelinase [11]; the ensuing generation of ceramide platforms causes pore-containing plasmalemmal invaginations, which are subsequently endocytosed [11], [12].

The second repair scenario - microvesicle shedding - is instrumental in the protection of neutrophils and endothelial cells from the trans-membrane pores formed by the membrane attack complex (MAC) of complement [13], [14], [15], [16]. Recently, we have shown that plasma membrane repair in cells, which were exposed to SLO, was accomplished by the shedding of toxin-bearing microvesicles [7], [10]. The isolation and physical removal of the toxin is triggered by the pore-induced rise in [Ca^2+^]_i_ and is effected by annexins; proteins which bind to phospholipids in a Ca^2+^-dependent manner, displaying membrane aggregating and fusogenic properties [3], [17].

The two modes of plasmalemmal repair differ in almost all aspects but they are not mutually exclusive: in human neutrophils, the MAC is removed both by endocytosis and microvesicle shedding [16], [18]. Whereas the shedding of the MAC predominates in neutrophils [16], endocytosis seems to be the primary route of MAC elimination in Ehrlich ascites tumor cells [19]. Thus, both the endocytic and the shedding route may simultaneously contribute to the removal of the pore-forming toxins; their relative contribution might differ between cell types or even within a particular cell type [16], [18].

Studies, which directly compared the contribution of the two mechanisms to the plasmalemmal repair of SLO pores, yielded inconsistent results. Whereas one study showed that microvesicle release but not lysosomal repair was responsible for the elimination of SLO pores in CHO and HeLa cells [9]; a second investigation, conducted on normal rat kidney (NRK), HeLa and HEK 293 cells came to the opposite conclusion [8].

The present study explores whether the extent and localization of the injury as well as the intrinsic features of a perforated cell might define a preferential route of plasmalemmal repair.

## Materials and Methods

### Cell Culture and Transfections

Human embryonic kidney cells (HEK 293) were maintained as previously described [12]. Human neuroblastoma (SH-SY5Y) cells were maintained in modified Eagle’s medium supplemented with 10% fetal bovine serum and 1% penicillin and streptomycin. The coding sequence of annexin A1 was cloned into the Living Colours Fluorescent protein vectors (Clontech, Mountain View, USA) following the PCR amplification from human bladder smooth muscle cDNA [20]. YFP (yellow-fluorescent protein), CFP (cyan-fluorescent protein), annexin A1-YFP or annexin A1-CFP were transiently expressed in target cells [20].

### Cell Lysis

Cell lysis was assessed by monitoring the irreversible elevation of intracellular [Ca^2+^] above 20 µM, using the permanent translocation of the calcium-sensitive protein annexin A1 from the cytoplasm to the plasma membrane as a read-out [21]. Annexin A1-transfected cells seeded on glass coverslips were mounted in a perfusion chamber at 25°C in Tyrode’s buffer (140 mM NaCl, 5 mM KCl, 1 mM MgCl_2_, 10 mM glucose, 10 mM HEPES; pH = 7.4) containing 2.5 mM CaCl_2_. At time-point = 0, the cells were challenged with 100 ng/ml (if not stated otherwise) SLO from *Streptococcus pyogenes* pre-activated with 20 mM DTT. When indicated, the cells were pre-incubated with either Jasplakinolide (Sigma-Aldrich; 100 nM, 60 min, 37°C), or Latrunculin A (Sigma-Aldrich; 5 µM, 60 min, 37°C), or Calpeptin (Merck-Calbiochem; 60 µM, 30 min, 37°C), or Vacuolin-1 (Sigma-Aldrich; 20 µM, 3 h, 37°C), or Y-27632 (Sigma-Aldrich; 50 µM, 3 h, 37°C). Translocation of annexin A1 was recorded in an Axiovert 200 M microscope with a laser scanning module LSM 510 META (Zeiss, Germany) using a ×63 oil immersion lens [7]. The images were analysed using the “Physiology evaluation” software package (Zeiss, Germany).

Annexin A1-positive microvesicles that were released in individual experiments were counted manually (every 5^th^ frame) in the recorded videos (70 frames = 434s) and are expressed as total number of microvesicles/total number of cells in each individual experiment.

### Fluorescence-activated Cell Sorting (FACS) and Western Blot Analysis

Confluent HEK 293 cells transfected with annexin A1-YFP were used for FACS analysis. Confluent non-transfected HEK 293 cells were used for Western Blotting. 1×10^7^ cells were used per experiment. To generate microvesicles, cells were washed 3 times with Tyrode's buffer containing 2.5 mM CaCl_2_ and challenged for 20 min with SLO (100 ng/ml) pre-activated with 20 mM DTT. When indicated, the cells were pre-incubated with either Jasplakinolide (Sigma-Aldrich; 100 nM, 60 min, 37°C), or Latrunculin A (Sigma-Aldrich; 5 µM, 60 min, 37°C), or Calpeptin (Merck-Calbiochem; 60 µM, 30 min, 37°C), or Vacuolin-1 (Sigma-Aldrich; 20 µM, 3 h, 37°C). The microvesicle-containing medium was collected, centrifuged first for 10 min at 1,500 g and then for 30 min at 130,000 g. The pellet containing microvesicles was resuspended in 2.5 mM CaCl_2_ Tyrode’s buffer and analysed by FACS (SORP LSRII, Becton Dickinson, NJ USA). The Flowjo program suite, version 9.2, was used for data analysis. For Western Blotting analysis the pellet containing microvesicles was resuspended in 20 µl loading buffer. Primary anti-annexin A1 antibody (Hybridoma EH17A) was from DSHB, University of Iowa. Secondary HRP-conjugated antibody was from GE Healthcare, UK.

### β-Hexosaminidase Assay

Cells were grown to 70% confluence. SLO in Tyrode’s buffer containing 2.5 mM Ca^2+^ was added for 15 min and culture supernatants were collected immediately thereafter. Cells lysed by sonication were used to estimate total cellular β-hexosaminidase (100%). Released β-hexosaminidase was measured in culture supernatants as described [22], [23]. Fluorescence was measured at 365 nm (ex)/450 nm (em) using a Gemini EM Fluorescence microplate reader.

## Results

### The Dynamics of the Subcortical Actin Cytoskeleton Affect the Release of Microvesicles

**Figure 1:**
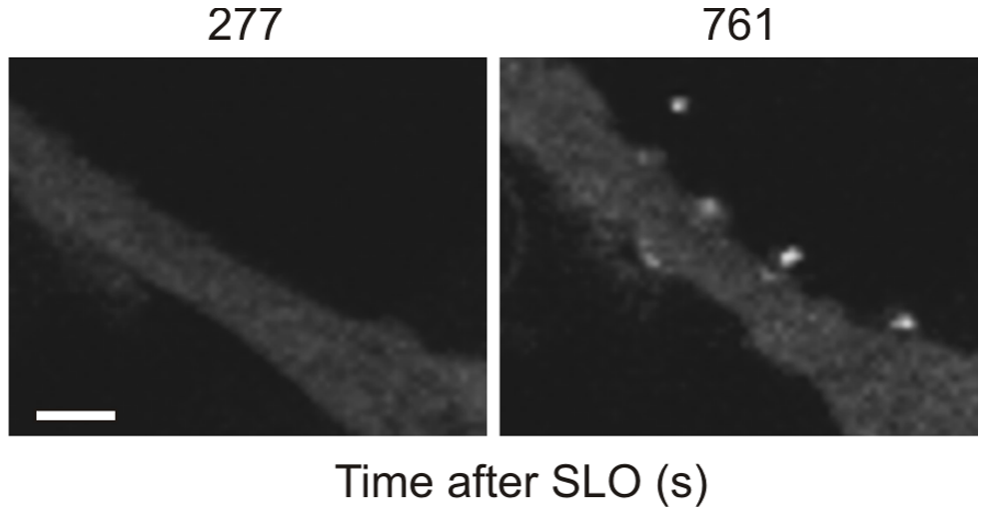
Plasmalemmal repair in SLO-damaged cells is accomplished by the expulsion of microvesicles. Shedding of annexin A1-rich microvesicles by SLO-treated, annexin A1-YFP-expressing HEK 293 cells was recorded by laser-scanning confocal microscopy. Magnification bar = 5 µm.

Recently we have shown that plasmalemmal repair in SLO-damaged HEK 293 cells is accomplished by the expulsion of annexin- and toxin-bearing microvesicles [7], [10]. Shedding of annexin A1-rich microvesicles by repaired HEK 293 cells that were treated with SLO/DTT is documented in Figure 1 and Video S1, whereas Video S2 shows that treatment with DTT alone did not induce microvesicle release.

Membrane tension, generated by the subcortical actin cytoskeleton, is a major factor that defines the rate of plasmalemmal resealing [6]. Thus, we investigated whether the protection against plasmalemmal injury brought about by the destabilization of the actin cytoskeleton [8], [24], [25] can be attributed to enhanced microvesicle release by SLO-damaged cells. Since significant differences in microvesicle counts were attributed to minor changes in experimental protocols in a number of studies (for critical assessment of the methodological approaches, currently used in microvesicle research, please see: [26], [27], [28]), in the present study, the amount of microvesicles, released under any experimental condition (specific treatment+SLO), were directly compared to the levels of microvesicles, released under the condition of SLO-alone treatment; in each individual experiment both protocols were always processed in parallel. Furthermore, to minimize an inevitable contribution of artifacts in the evaluation of our data, two independent approaches were used to analyze microvesicle release.

**Figure 2:**
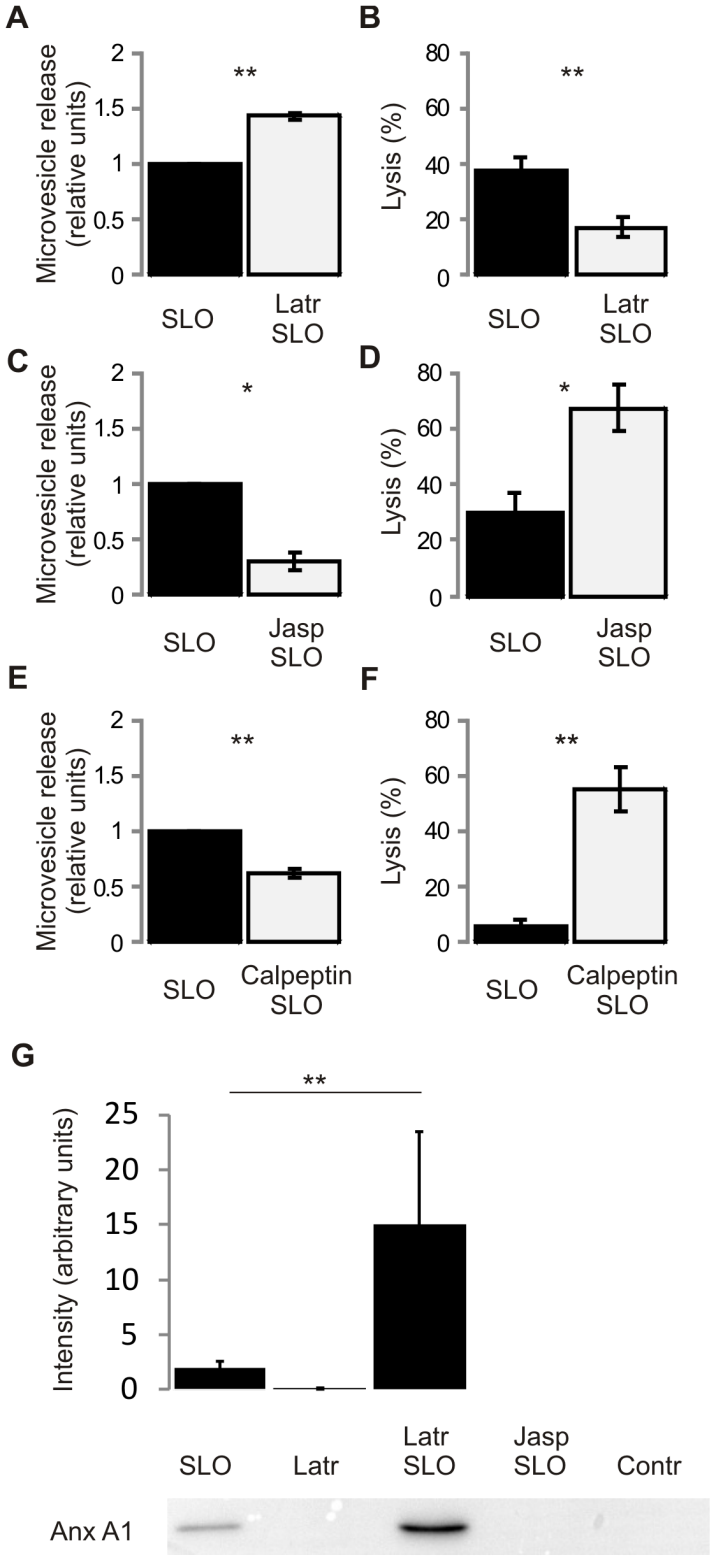
Destabilization of the cortical cytoskeleton enhances microvesicle release by SLO-damaged cells and potentiates their survival. (**A**) Enhanced release of microvesicles by SLO-damaged cells, which were treated with latrunculin A. (**B**) Latrunculin A protects HEK 293 cells from SLO-induced cell lysis. (**C**) Diminished release of microvesicles by SLO-damaged cells which were treated with jasplakinolide. (**D**) Treatment with jasplakinolide results in increased cell lysis. (**E**) Calpeptin reduces microvesicle release by SLO-damaged cells. (**F**) SLO induces an increased rate of lysis in calpeptin-treated cells. **p<0.001, *p<0.01. (G) Amounts of annexin A1 shed within microvesicles were analyzed by Western Blotting in culture supernatants of SLO-treated cells pre-treated with either latrunculin (Latr/SLO), jasplakinolide (Jasp/SLO), cells treated with latrunculin without SLO treatment (Latr) or cells treated with DTT only (Contr). **p<0.001.

Latrunculin A was used to destabilize the actin cytoskeleton [29]. FACS analysis of microvesicle contents in the supernatants of SLO-damaged cells revealed enhanced microvesicle shedding by cells treated with latrunculin A (Fig. 2A). In accordance with previous studies [8], destabilization of the actin cortex protected HEK 293 cells from SLO induced cell death (Fig. 2B). In a reciprocal experiment, HEK 293 cells were treated with jasplakinolide to stabilize the actin cytoskeleton [30]. FACS analysis of culture supernatants demonstrated a significantly lower number of microvesicles released by the jasplakinolide/SLO-treated cells compared to the SLO-treated cells (Fig. 2C). The reduction in microvesicle shedding by jasplakinolide-treated cells was accompanied by an increase in their lysis (Fig. 2D). *In vivo,* the destabilization of the cortical actin cytoskeleton, which is required for a successful repair of plasmalemmal lesions, is achieved by calpains, a family of non-lysosomal Ca^2+^-dependent proteases [31]. Calpeptin, a cell-permeable inhibitor of calpain [13], [32], [33], reduced microvesicle shedding by SLO-damaged cells (Fig. 2E). Consequently, SLO induced an increased rate of lysis in calpeptin-treated cells (Fig. 2F). Neither cell lysis nor microvesicle shedding was observed in control experiments in which cells were incubated with DTT in the absence of SLO.

These findings were further validated in an independent experiment. As documented in Figure 1A and Videos S1 and S2, annexin A1 is exclusively released by shedding HEK 293 cells. Therefore, the amounts of annexin A1 released by SLO-treated cells were analyzed by Western Blotting in culture supernatants of cells pre-treated with either latrunculin or jasplakinolide (Fig. 2G). Destabilization of actin cytoskeleton by latrunculin increased shedding of annexin A1 by SLO-damaged cells, whereas the stabilization of the actin cytoskeleton reduced its amounts in the culture medium below the detection limit of the method. No shedding of annexin A1 was observed in cells treated with either latrunculin alone or DTT alone (control).

Since destabilization of the cortical cytoskeleton also facilitates lysosomal repair [8], it appears that both lysosomal repair and microvesicle shedding are similarly affected by the flexibility of the plasmalemmal lipid bilayer, which itself critically depends on the remodeling of the underlying actin cytoskeleton. Also, the two mechanisms are triggered by a pore-induced elevation in [Ca^2+^]_i_ [8], [10]. These similarities make it even more important to comprehend the specific features that guide a damaged cell in its choice of a suitable repair strategy.

### Repair of Limited Plasmalemmal Damage by Pinpoint Microvesicle Shedding

**Figure 3:**
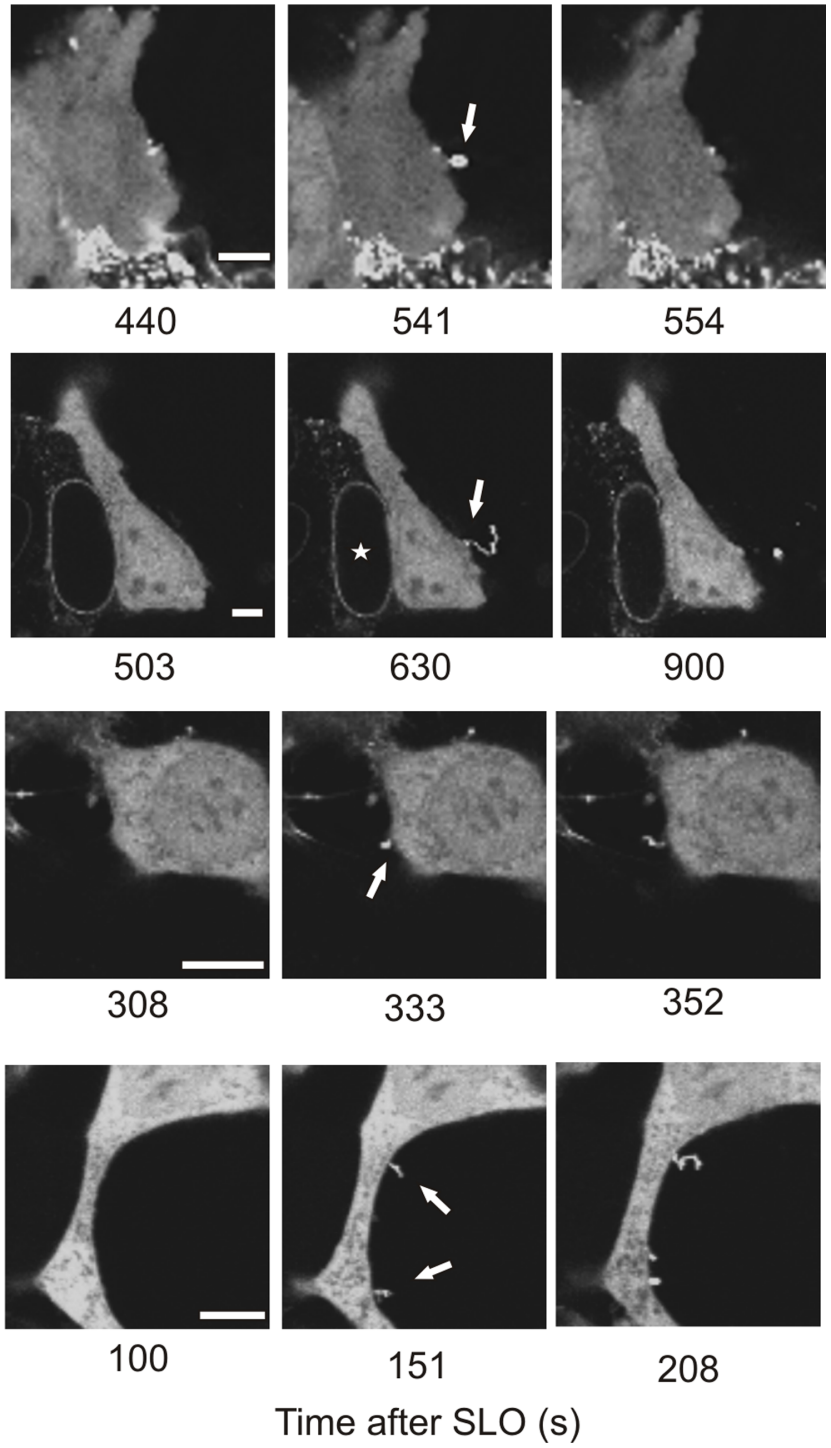
Pinpoint shedding of microvesicles. Two HEK 293 and two SH-SY5Y cells transfected with annexin A1-YFP are shown. SLO-pores are quarantined within compact plasmalemmal regions or within thin outward protrusions (arrows). An asterisk denotes the nucleus of a lysed cell. Magnification bars = 5 µm.

Plasma membrane perforation by a toxin pore manifests itself in the elevation of [Ca^2+^]_i_. As a result, the Ca^2+^-dependent binding to charged phospholipids drives the translocation of annexins from the cytoplasm to the plasmalemma [3], [7], [10]. After pore formation, the damaged membrane is quarantined by the annexins [3], [10] within compact plasmalemmal spots or within thin outward protrusions and eventually shed into the extracellular milieu (Fig. 3, Videos S1, S3, S4). Since the binding of annexins to the plasma membrane occurs exclusively at elevated [Ca^2+^]_i_ [3], [17], [20], the pinpoint accumulation of annexin A1 at the damaged plasmalemma reveals that the pore-induced Ca^2+^ entry, which could distort the otherwise tightly regulated Ca^2+^ homeostasis, remains spatially confined.

Thus, annexin-mediated microvesicle shedding seems to be optimal for the early repair of individual toxin pores allowing the survival of damaged cells with minimal detrimental consequences.

### The Plasmalemmal Repair of Toxin Pores Occurs in Lysosome-free Intracellular Compartments

**Figure 4:**
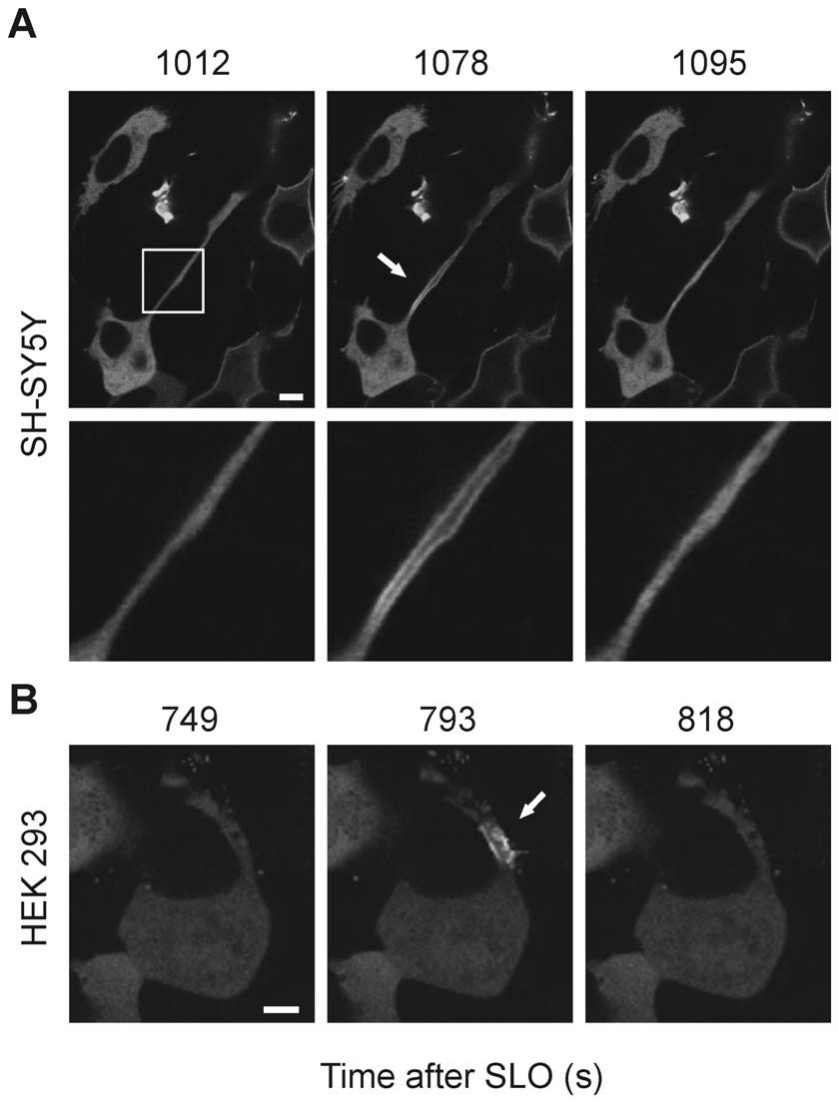
The plasmalemmal repair of SLO pores occurs in lysosome-free cellular protrusions. (**A,B**) SLO-induced plasmalemmal translocation/cytoplasmic back-translocation of annexin A1-YFP marks the successful elimination of the SLO-pores in (**A**) a neurite of a SH-SY5Y cell or in (**B**) a cytoplasmic protrusion of a HEK 293 cell. Arrows denote the plasmalemmal translocation of annexin A1 (plasmalemmal permeabilization). Magnified images of the squared region are shown in (**A**). Magnification bars = 5 µm.

The lysosomal-fusion mode of plasmalemmal repair is restricted to the cell body where lysosomes are localized, and thus is not likely to happen at stable cellular protrusions such as axons or dendrites which are usually free of intracellular organelles [34]. Such protrusions, which are located at the body’s periphery, are most likely the very first cellular components to encounter bacterial toxins. Figure 4A (Video S5) demonstrates that the SLO-pores can be successfully eliminated from the plasmalemma of the elongated, thin neurites of SH-SY5Y neuroblastoma cells. The permeabilization manifests itself in the translocation of annexin A1 within the neurite (Fig.4A, time = 1078 s). The translocation of the annexin follows the propagation of the pore-induced Ca^2+^-wave, which ceases before reaching the cell body, whereupon annexin returns to the cytoplasm of the neurite (Fig.4A, time = 1095 s, Video S5). Since the annexins can relocate to the cytoplasm only after the toxin pores have been eliminated and [Ca^2+^]_i_ homeostasis has been re-established, the back-translocation of the annexin marks the successful elimination of the SLO-pores. Figure 4B documents plasmalemmal repair occurring in a protrusion of a HEK 293 cell.

**Figure 5:**
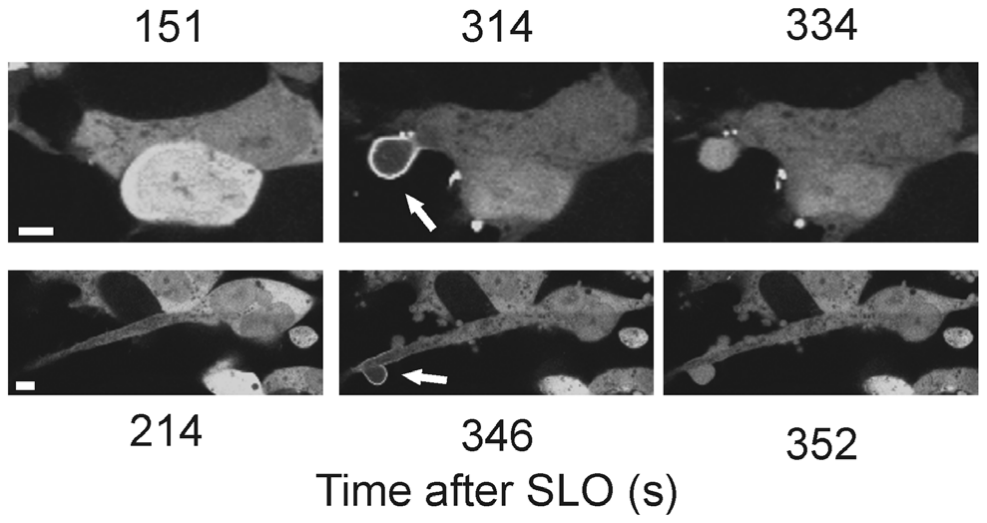
The plasmalemmal repair of SLO pores occurs in lysosome-free blebs. SLO-induced plasmalemmal translocation/cytoplasmic back-translocation of annexin A1-YFP marks the successful elimination of the SLO-pores in blebs of HEK 293 cells. Arrows denote the plasmalemmal translocation of annexin A1 (plasmalemmal permeabilization). Magnification bars = 5 µm.

The intracellular trafficking of bulky lysosomes is expected to be relatively slow. Thus, it is unlikely that a sufficient number of lysosomes will be locally available for the plasmalemmal repair in cells which undergo a continuous rearrangement of their shape. Blebs are cytoplasmic spherical protrusions, sprouted by cells during stressful conditions, which are connected to the cell body by a thin neck, but which are free of intracellular organelles [35]. A pore within a bleb manifests itself in the elevation of intra-bleb [Ca^2+^]_i_ followed by plasmalemmal translocation of annexin A1 within the perforated bleb (Fig. 5). Back-translocation of annexin A1 into the cytoplasm reveals the successful elimination of the SLO-pores from the plasmalemma of lysosome-free blebs (Fig.5; Video S6). In the experiments described here, the pore-induced [Ca^2+^]_i_ elevation within the cell body remains low (annexin remains in the cytoplasm). Thus, SLO-pores occurring within the lysosome-free blebs can successfully be eliminated even before the danger signal ([Ca^2+^]_i_-elevation) reaches the cell body.

Collectively, these results demonstrate that plasmalemmal repair occurs in regions of the cell in which lysosomes are not available. It is effected, most likely, via annexin-driven microvesicle shedding since the carriers for this type of repair are cytoplasmic proteins, which are expressed throughout the cell including its cytoplasmic protrusions, and can also rapidly diffuse into newly formed cellular compartments.

### Extensive Toxin-induced Damage is Accompanied by Self-inflicted Mechanical Damage

**Figure 6:**
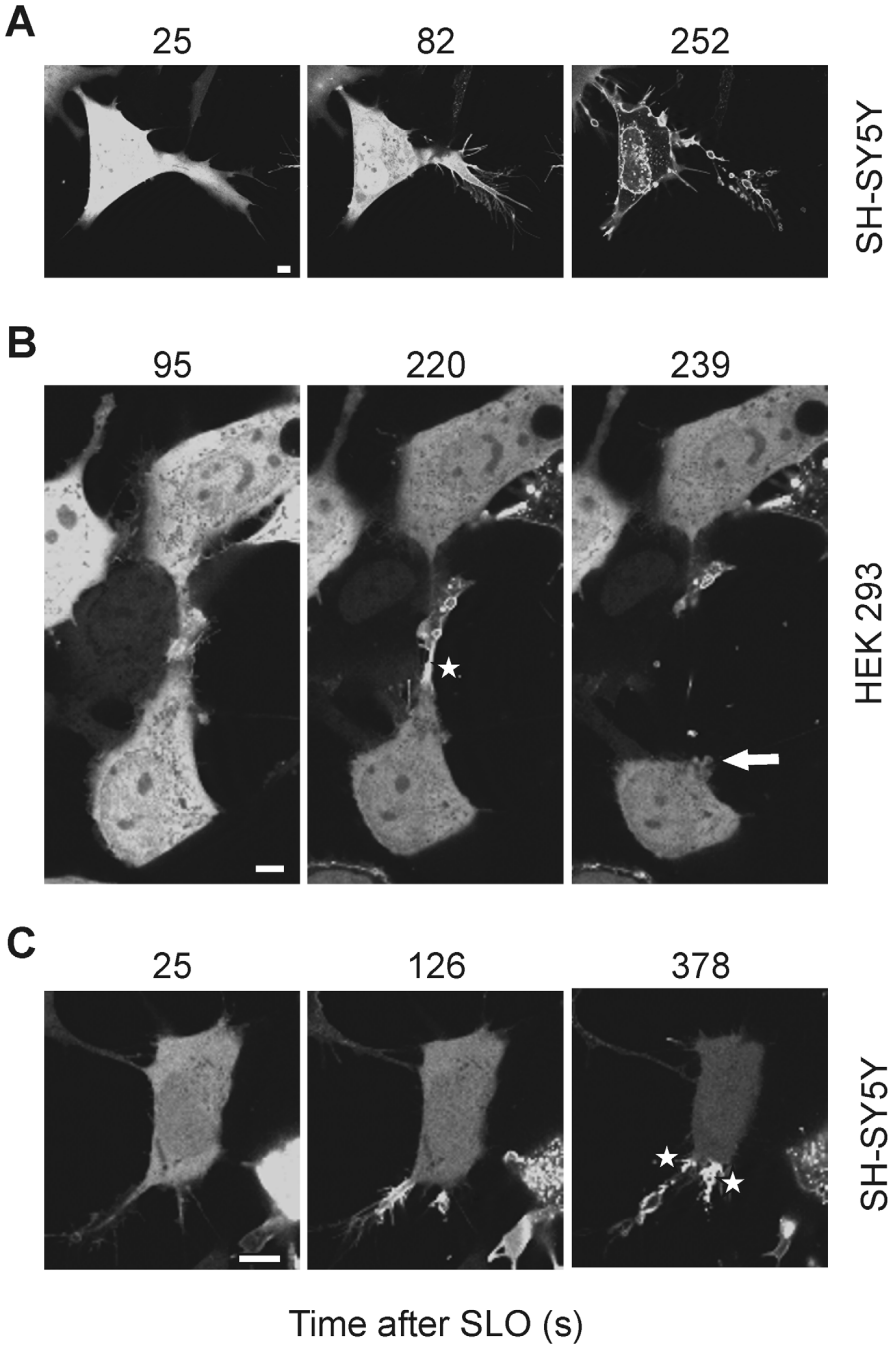
Self-inflicted mechanical damage in SLO-perforated cells. (**A–C**) The SLO-perforated protrusions of SH-SY5Y or HEK 293 cells are wrenched apart by mechanical forces. (**A**) The global translocation of annexin A1-YFP manifests cell lysis. (**B,C**) The cytoplasmic localization of annexin A1-YFP within the cell body is evidence of successful resealing of the plasmalemma. The arrow in (**B**) points at the resealed base of the destroyed protrusion. The asterisks in (B,**C**) denote regions of clear demarcation between permeabilized (plasmalemmal localization of annexin A1-YFP) and non-permeabilized (cytoplasmic localization of annexin A1-YFP) cellular compartments. Magnification bars = 5 µm.

Extensive formation of toxin-pores does not only induce a localized elevation of [Ca^2+^]_i_, which is needed to trigger plasmalemmal repair, but can also push the global [Ca^2+^]_i_ above the threshold, required for a prolonged activation of cellular contractile elements. The following uncontrolled contraction can lead to substantial mechanical damage of the plasmalemma. Figure 6A (Video S7) shows a toxin-perforated SH-SY5Y cell. The toxin-induced Ca^2+^-elevation leads to a contraction of the cell’s protrusion, which is tightly attached to the substratum. As a result, the protrusion is wrenched apart by the mechanical forces. The remains of the protrusion’s plasmalemma, which remains attached to the substratum, are highlighted by bound annexin A1 (Fig. 6A, Video S7). The global translocation of the annexin within the cell body and its nucleus manifests cell lysis. However, even such a massive, self-inflicted mechanical damage of the plasmalemma can be repaired. Figure 6B (Video S8) shows two HEK 293 cells which are connected by long cytoplasmic protrusions. An initial perforation occurring within a protrusion leads to a local translocation of annexin A1 followed by contraction and rupture of the protrusion of the lower cell. Its distal part remains attached to the protrusion of the upper cell whereas the rest of the protrusion is ripped apart by the strength of the contraction. However, the plasmalemma at the base of the protrusion is able to reform (Fig. 6B, arrow) and the cytoplasmic localization of annexin A1 within the cell body is the evidence of successful resealing of the plasmalemma (Fig. 6B). Likewise, the mechanical lesions occurring in a SLO-perforated SH-SY5Y cell are efficiently repaired (Fig. 6C; Video S9).

### Inhibition of Myosin-driven Contraction does not Increase Survival of Toxin-damaged SH-SY5Y Cells or HEK 293 Cells

Since an initial perforation of the plasmalemma by pore-forming toxins can lead to massive secondary mechanical damage, inflicted by the activated contractile machinery, we next probed whether lysis of toxin-perforated cells might be prevented by inhibiting myosin contraction with Y-27632 [36], [37].

**Figure 7:**
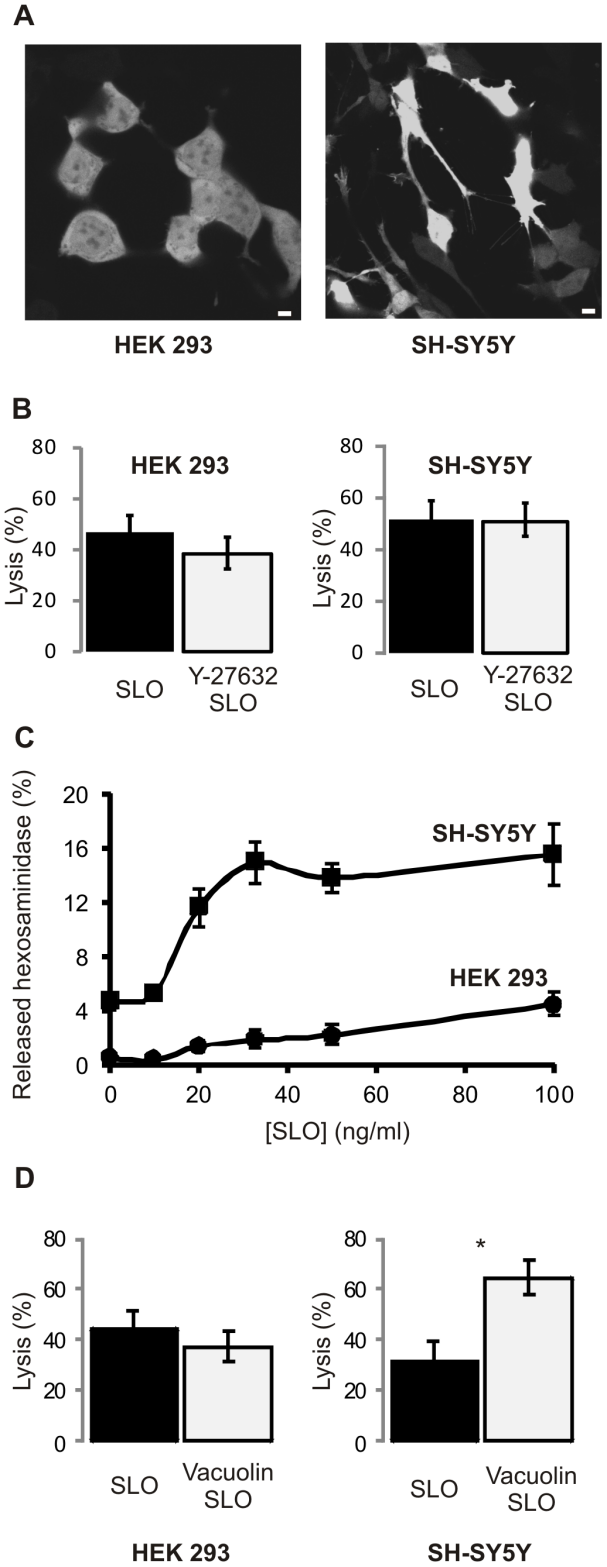
SLO-perforation inflicts mechanical damage and triggers lysosomal fusion. (**A**) HEK 293 cells do not adhere extensively to the substratum at the cellular periphery while SH-SY5Y cells are firmly attached by multiple protrusions. Magnification bars = 2 µm. (**B**) Inhibition of myosin contraction does not protect from SLO induced lysis. (**C**) Lysosomal exocytosis (β-hexosaminidase release) after SLO-injury is more pronounced in SH-SY5Y cells compared to HEK 293 cells. (**D**) Vacuolin-1 does not increase the SLO-induced lysis in HEK 293 cells. In contrast, Vacuolin-1-treated SH-SY5Y cells are more prone to the SLO-induced lysis. *p<0.01.

HEK 293 cells retain their bulky geometry when grown on glass and do not form extensive adherence contacts with the substratum at the cellular periphery (Fig. 7A). Since in HEK 293 cells the contraction-induced damage events are relatively rare (Video S10) and occur mostly in protrusions of adjacent cells which are linked with each other (Fig. 6B), the inhibition of myosin contraction did not lead to increased survival of these cells (Fig. 7B).

In contrast, SH-SY5Y cells spread on glass coverslips by sprouting multiple protrusions, which adhere vividly to the support (Fig.7A) and are prone to secondary mechanical injury when they contract (Video S11). Most surprisingly, the inhibition of myosin contraction also did not protect these cells from SLO-induced lysis (Fig. 7B). Thus, mechanical damage was not the defining factor in the lysis of SLO-perforated SH-SY5Y cells, most likely, because such injuries were efficiently repaired.

### Lysosomal Fusion is Essential for the Resealing of SH-SY5Y Cells but not that of HEK 293 Cells

Annexins seem to be instrumental in quarantining mechanical injuries (Fig. 6B,C, asterisks). However, since the resealing of extended mechanical lesions requires a deposition of additional membranous material [6], the pinpoint repair action of the annexin-driven microvesicle release is not compatible with this mode of repair. Instead, a lysosomal patch is much better suited for the repair of such extensive lesions [6].

Correspondingly, lysosomal fusion accompanied by the release of lysosomal β-hexosaminidase [22], [23] was more prominent in SLO-treated SH-SY5Y cells compared to HEK 293 cells (Fig. 7C). Vacuolin-1, which blocks the Ca^2+^-dependent exocytosis of lysosomes and thus prevents their fusion with the plasma membrane [22], [38], did not increase the SLO-induced lysis of HEK 293 cells (Fig. 7D). In contrast, vacuolin-1 treatment significantly enhanced the SLO-induced lysis of SH-SY5Y cells (Fig. 7D). Thus, whereas myosin contraction and the concomitant mechanical injuries themselves did not increase the rate of SLO-induced SH-SY5Y cell lysis, the inhibition of repair mechanisms that are responsible for the elimination of such injuries did.

### Microvesicle Shedding and Lysosomal Repair Complement each other during Plasmalemmal Repair in SH-SY5Y Cells

Microvesicle shedding and repair of self-inflicted mechanical lesions were observed in toxin-damaged HEK 293 and SH-SY5Y cells. As discussed above, the self-inflicted mechanical damage was only a minor occurrence in HEK 293 cells. Correspondingly, the inhibition of lysosomal repair did not significantly affect the overall outcome of plasmalemmal repair (Fig.7). Thus microvesicle shedding was largely responsible for the repair of the toxin-damaged plasmalemma in these cells. In contrast, the self-inflicted mechanical damage was more frequent in SH-SY5Y cells contributing significantly to the overall outcome of plasmalemmal repair (Fig.7).

**Figure 8:**
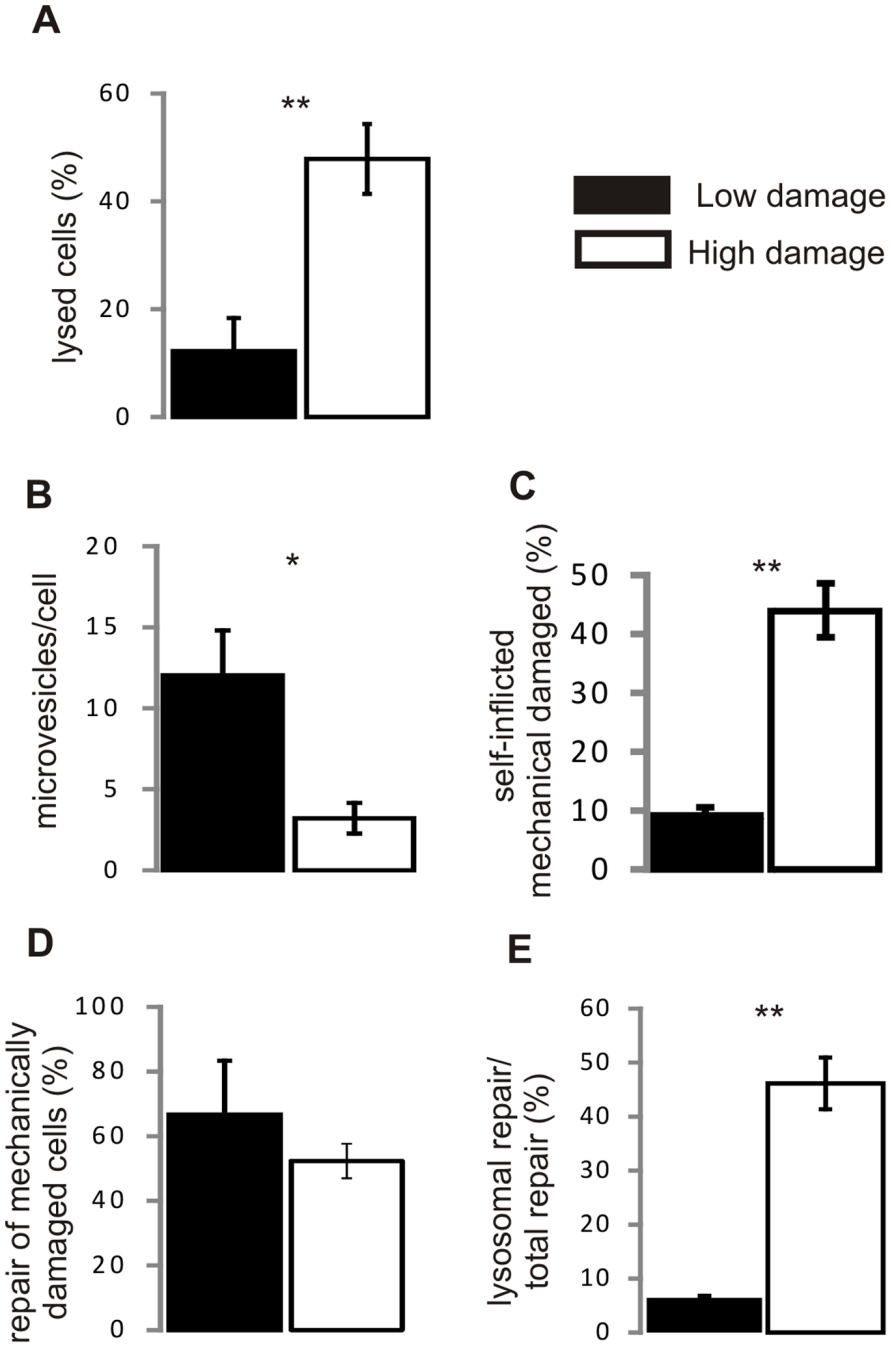
Microvesicle shedding is responsible for the initial elimination of toxin pores whereas lysosomal repair mends secondary, self-inflicted mechanical injuries. (**A**) Individual experiments in which SH-SY5Y cells either repaired efficiently (low damage; 72 cells, 3 independent experiments) or suffered from extensive plasmalemmal damage (high damage; 66 cells, 3 independent experiments) were analyzed for: (**B**) amount of microvesicles released per cell, (**C**) percentage of cells that suffered from secondary, self-inflicted mechanical damage (100% = total number of cells in each individual experiment), (**D**) percentage of cells that recovered after self-inflicted mechanical damage (100% = number of mechanically-damaged cells in each individual experiment), (**E**) contribution of lysosomal repair to total repair (100% = total number of repaired cells in each individual experiment). **p<0.001, *p<0.01.

Our data suggest that self-inflicted mechanical damage, which requires lysosomal repair, might occur preferentially in heavily-damaged SH-SY5Y cells that initially failed to eliminate SLO-pores by microvesicle shedding. To determine the relative contribution of microvesicle shedding and lysosomal repair to total plasmalemmal repair we evaluated the contribution of either repair mechanism in individual experiments in which SH-SY5Y cells **a)** repaired efficiently (lysis in any individual experiment was below 20%) or **b)** suffered from extensive plasmalemmal damage (lysis in any individual experiment was above 40%) (Fig.8A). Figure 8B shows that during efficient repair (low damage), significantly more microvesicles were shed by individual cells compared to the experiments in which cells failed to repair efficiently (high damage). Correspondingly, microvesicle shedding was the predominant repair mechanism in the efficiently repaired cells since only 9±1.5% of the cells suffered from secondary, self-inflicted mechanical damage at these conditions (Fig. 8C). In contrast, in the experiments in which cells failed to repair efficiently, 44±4.7% were damaged mechanically (Fig. 8C). No significant difference between the two conditions was observed in the repair of cells that suffered from the self-inflicted mechanical damage (Fig. 8D). Whereas at the low damage conditions 4 out of 7 mechanically-damaged cells were able to repair (66.6±16.6%), 15 out of 29 mechanically damaged cells repaired at the high damage conditions (52.3±5.4%) suggesting that once the plasmalemmal damage was acute enough to induce self-inflicted mechanical damage, the repair of individual mechanically damaged cells was effected with the same efficiency by the same mechanism (lysosomal repair). Thus, the difference in total plasmalemmal repair observed between the two experimental conditions (Fig, 8A) was largely defined by the efficiency of the initial plasmalemmal repair effected by microvesicle shedding. Overall, lysosomal repair was instrumental in 6.4±0.65% of cells that repaired at low-damage conditions and in 46±4.8% of cells that repaired at the conditions of high damage (Fig, 8E).

## Discussion

We show that both microvesicle shedding and lysosomal repair are instrumental in the restoration of membrane barrier function following plasmalemmal permeabilization by bacterial pore-forming toxins.

Molecular mechanisms that govern lysosomal repair are well characterized especially during repair of mechanical injuries [4], [5], [6], whereas those effecting microvesicle shedding are not yet established in detail. In particular, the mechanisms responsible for the formation of the initial membrane evaginations, which are required for the outward vesiculation and the processes that govern the pinch-off of toxin-bearing microparticles are not well characterized. It is conceivable that the initial outward curvature is brought about by the lipolytic processing of plasmalemmal constituents [39], whereas the fusogenic activity of the annexins might be responsible for the microparticle release [3].

Here we provide evidence that additional supportive or alternative mechanisms might be at play. Our present results call attention to the formation of thin outward protrusions during the process of the elimination of individual SLO-pores by microvesicle shedding, which resemble tunneling nanotubes,- membrane nanostructures that are formed by a variety of cells under stress conditions [40], [41], [42]. Nanotubes can also dilate into spherical structures, similar to those described in the present report, that may pinch off from- or remain attached to a mother cell [43]. Serving, presumably, as the means of direct cell-to-cell communication, the nanotubes have a diameter of 180–380 nm, are not tethered to the substratum and might form direct seamless contacts with the neighboring cells [41]. Whereas the stability of membrane nanotubes *in vivo* is reinforced by an actin cytoskeleton, their formation is governed exclusively by the thermodynamic properties of the membrane lipid bilayer [43]. Thus, lateral redistribution of membrane constituents, brought about by the formation of a toxin pore, might be sufficient to induce nanosized membrane buds, which elongate into nanotubes and are pinched off from the membrane to become micro(nano)vesicles [43]. Since, the formation of nanotubes and their vesiculation/detachment from the mother vesicles have been documented in artificial giant phospholipid vesicles [44], these processes might occur spontaneously after destabilization of the lipid bilayer by the toxin-pore formation and presumably require no input from other cellular constituents.

We show that both microvesicle shedding and lysosomal repair, which are triggered by the same mechanism (pore-induced elevation in [Ca^2+^]_i_), are similarly affected by the flexibility of the plasmalemmal lipid bilayer. Thus identical basic mechanisms apply for both modes of pore elimination; the differences transpire in more subtle details, and lead to wide-ranging consequences.

Different tasks performed by the two modes of plasmalemmal repair are, most likely, defined by their physical carriers (proteins versus organelles) and dependent on (i) intrinsic properties of a cell (e.g. capability to induce secondary, self-inflicted mechanical damage), (ii) morphology of a cell and (iii) the repair-induced changes in cellular homeostasis.

Whereas spatially well-confined toxin lesions can be swiftly eliminated with minimal detrimental consequences for a cell, an extensive formation of toxin-pores is often accompanied by massive mechanical damage. Our data suggest that annexins are best positioned for the instant repair of individual membrane lesions via microvesicle shedding, whereas lysosomal fusion is indispensable for the repair of concomitant mechanical injuries.

Whereas the fusion of bulky lysosomes is restricted to the plasmalemma of the cell body, the shedding of microvesicles, which is effected by the relatively mobile proteins of the annexin family can operate also at the cellular periphery. Additionally, lysosomal repair of toxin-induced lesions, which are difficult to access, might be achieved by their conversion into mechanical ones: the total destruction of the pore-bearing, lysosome-free protrusions results in the elimination of the toxin-pore by its release into the extracellular milieu. Simultaneously, a mechanically-inflicted lesion is created in the vicinity of the lysosome-rich cell body where lysosomes are available for plasmalemmal repair. Thus, when microvesicle shedding fails to eliminate SLO-pores at the cellular periphery, a cell enters a “lizard tail” mode of action: in order to avoid its total destruction, it sacrifices the damaged peripheral regions. A similar function can be ascribed to plasmalemmal blebbing [21]. Both blebbing and the self-inflicted destruction of perforated peripheral components are prompted by actin-myosin contraction. The role of myosin-driven contraction in plasmalemmal repair is emphasized by the inability to repair injuries, in which myosin is inhibited by blebbistatin [21].

The need for repair at two different levels, involving microvesicle shedding and lysosomal fusion might be also defined by additional hazards, which occur after the successful resealing of plasmalemmal lesions. During the repair of extensive damage, cells experience a prolonged and excessive elevation in [Ca^2+^]_i_. Ca^2+^ is a critical second messenger, which is involved in the regulation of a multitude of cellular processes which necessitates tight control of its intracellular concentration [45]. The uncontrolled elevation in [Ca^2+^]_i_ during plasmalemmal repair might lead to a homeostatic imbalance, hyper- or de-activation of vital cellular signalling pathways and irreversible changes in their gene expression pattern [18], [46], [47]. Thus, the effective repair after an extensive injury might lead to even more disastrous long term consequences than the lysis of a damaged cell.

Therefore, perforated cells are confronted with three tasks: their lysis must be prevented; repaired cells, which were extensively damaged, must be eliminated; and slightly damaged cells must be re-vitalized. During repair by microvesicle shedding, the toxin pore is immediately quarantined by the annexins; the pore is expelled into the extracellular milieu with minimal detrimental consequences allowing the cell to return to its normal state of function. In contrast, lysosome-plasmalemmal fusion is accompanied by major biochemical and structural changes within the plasmalemma. An exposure of the sphingomyelin-rich outer leaflet of the plasmalemmal lipid bilayer to the lysosomal acid sphingomyelinase leads to the formation of the pro-apoptotic sphingolipid ceramide [11]. The pro-apoptotic action of ceramide is greatly potentiated by its self-association within plasmalemmal ceramide platforms [48], [49]. The formation of ceramide platforms in cells, which were heavily damaged by SLO, has been reported earlier [12] and the inward plasmalemmal budding caused by their assembly following lysosome-plasmalemmal fusion is a prerequisite for the endocytosis of pore-bearing membranes [11]. Whereas multiple pathways are involved in ceramide-induced apoptosis, the very formation of ceramide platforms is presumably sufficient to trigger apoptosis. The inward budding and internalization of ceramide platforms is followed by their fusion with mitochondria and permeabilization of the mitochondrial outer membrane [50], [51]. Thus, plasmalemmal repair by lysosomal fusion appears to be perfectly suited for the apoptotic elimination of repaired cells that suffer from a surfeit of Ca^2+^.

Our data suggest that both microvesicle shedding and lysosomal fusion are employed by toxin-damaged cells to enable an intrinsic resealing of a lipid bilayer - a fundamental process, which is facilitated by the destabilization of the cortical cytoskeleton and is mediated by the spontaneous reorganization of plasmalemmal lipid constituents into their thermodynamically most favorable state. Whereas the pinpoint action of microvesicle shedding is optimal for the early elimination of toxin pores allowing the survival of damaged cells, the lysosomal fusion is best suited for the repair of extended secondary self-inflicted mechanical injuries.

## Supporting Information

Video S1 **Video S1 shows the release of microvesicles by SLO-permeabilized HEK 293 cells.** HEK 293 cells, transfected with annexin A1-YFP, were challenged with SLO/DTT. The movie (time-lapse mode) spans 2643 s.
(MOV)

Video S2 **Video S2 shows that non-permeabilized HEK 293 cells do not release microvesicles.** HEK 293 cells, transfected with annexin A1-YFP, were challenged with DTT alone. The movie (time-lapse mode) spans 2643 s.
(MOV)

Video S3 **Video S3 shows budding and release of an annexin A1-enriched microvesicle by a SLO-treated HEK 293 cell.** HEK 293 cells, transfected with annexin A1-YFP, were challenged with SLO. Partial view of one cell. The movie (time-lapse mode) spans 157 s.
(MOV)

Video S4 **Video S4 shows the formation of an annexin A1-enriched plasmalemmal protrusion, followed by release of microvesicles in a SLO-treated HEK 293 cell.** HEK 293 cells, transfected with annexin A1-YFP, were challenged with SLO. The movie (time-lapse mode) spans 403 s.
(MOV)

Video S5 **Video S5 shows a plasmalemmal translocation-cytoplasmic back-translocation of annexin A1 localized within a neurite of a SLO-treated SH-SY5Y cell.** SH-SY5Y cells, transfected with annexin A1-YFP, were challenged with SLO. The movie (time-lapse mode) spans 124 s.
(MOV)

Video S6 **Video S6 shows a plasmalemmal translocation-cytoplasmic back-translocation of annexin A1 localized within a bleb of a SLO-treated HEK 293 cell.** Hek 293cells, transfected with annexin A1-YFP, were challenged with SLO. The movie (time-lapse mode) spans 201 s.
(MOV)

Video S7 **Video S7 shows a plasmalemmal translocation of annexin A1 localized within a protrusion of a SLO-treated SH-SY5Y cell, followed by contraction and rupture of the protrusion.** Note the plasmalemmal localization of annexin A1 within the cell body of the damaged cell. SH-SY5Y cells, transfected with annexin A1-YFP, were challenged with SLO. The movie (time-lapse mode) spans 258 s.
(MOV)

Video S8 **Video S8 shows a plasmalemmal translocation of annexin A1 localized initially within a protrusion of a SLO-treated HEK 293 cell, followed by contraction and rupture of the protrusion.** Note the cytoplasmic localization of annexin A1 within the cell body of the damaged cell. HEK 293 cells, transfected with annexin A1-YFP, were challenged with SLO. The movie (time-lapse mode) spans 844 s.
(MOV)

Video S9 **Video S9 shows a plasmalemmal translocation of annexin A1 localized within protrusions of a SLO-treated SH-SY5Y cell, followed by contraction and rupture of the protrusions.** Note the cytoplasmic localization of annexin A1 within the cell body of the damaged cell. SH-SY5Y cells, transfected with annexin A1-YFP, were challenged with SLO. The movie (time-lapse mode) spans 415 s
(MOV)

Video S10 **Video S10 shows that SLO-induced damage does not induce significant contraction of HEK 293 cells.** HEK 293 cells, transfected with annexin A1-YFP, were challenged with SLO. The movie (time-lapse mode) spans 938 s
(MOV)

Video S11 **Video S11 shows that SLO-induced damage is accompanied by massive contraction of extended protrusions of SH-SY5Y cells.** SH-SY5Y cells, transfected with annexin A1-YFP, were challenged with SLO. The movie (time-lapse mode) spans 938 s
(MOV)
